# Assembling Neurospheres: Dynamics of Neural Progenitor/Stem Cell Aggregation Probed Using an Optical Trap

**DOI:** 10.1371/journal.pone.0038613

**Published:** 2012-06-05

**Authors:** Uma Ladiwala, Himanish Basu, Deepak Mathur

**Affiliations:** 1 UM-DAE Centre for Excellence in Basic Science, University of Mumbai, Kalina Campus, Mumbai, India; 2 Tata Institute of Fundamental Research, Mumbai, India; University of Ottawa, Canada

## Abstract

Optical trapping (tweezing) has been used in conjunction with fluid flow technology to dissect the mechanics and spatio-temporal dynamics of how neural progenitor/stem cells (NSCs) adhere and aggregate. Hitherto unavailable information has been obtained on the most probable minimum time (∼5 s) and most probable minimum distance of approach (4–6 µm) required for irreversible adhesion of proximate cells to occur. Our experiments also allow us to study and quantify the spatial characteristics of filopodial- and membrane-mediated adhesion, and to probe the functional dynamics of NSCs to quantify a lower limit of the adhesive force by which NSCs aggregate (∼18 pN). Our findings, which we also validate by computational modeling, have important implications for the neurosphere assay: once aggregated, neurospheres cannot disassemble merely by being subjected to shaking or by thermal effects. Our findings provide quantitative affirmation to the notion that the neurosphere assay may not be a valid measure of clonality and “stemness”. Post-adhesion dynamics were also studied and oscillatory motion in filopodia-mediated adhesion was observed. Furthermore, we have also explored the effect of the removal of calcium ions: both filopodia-mediated as well as membrane-membrane adhesion were inhibited. On the other hand, F-actin disrupted the dynamics of such adhesion events such that filopodia-mediated adhesion was inhibited but not membrane-membrane adhesion.

## Introduction

Stem cells are found in many tissues; they possess the unique ability to self-renew and differentiate into multiple cell types, properties that enable them to play a vital role in the maintenance of tissue integrity and homeostasis as also in repair subsequent to tissue damage. Their therapeutic potential has evinced tremendous contemporary interest and has resulted in numerous studies on various aspects of their dynamics, function and regulation, both in normal and pathological conditions.

The study of neural stem cell/progenitor (NSC) regulation, function and dynamics requires experimental methodologies that can reliably identify these cells and their progeny. The lack of specific phenotypic cell expression markers and access to fairly pure populations of stem cells has necessitated the use of functional assays to study neural stem cells *in vitro* and their potential to be evaluated by transplantation *in vivo* [1].

Stem cells and their progenitors are typically cultured *in vitro* either as dissociated two-dimensional adherent monolayers or three-dimensional neurospheres in suspension. Neurospheres are a tissue culture phenomenon represented by the formation of spheroid clusters *in vitro* when mitotic cells from the developing and/or adult mammalian central nervous system (CNS) are typically placed in serum-free medium on a non-adhesive substrate and exposed to mitogens. The quantification and characterization of these floating aggregates of cells has been used to define and measure stem cell-like behavior. This'neurosphere assay' has, over the years, assumed considerable importance [1]–[3]. It has been utilized to determine whether a cell is “stem-like” or not, based upon different neurosphere characteristics: (i) the number of neurospheres has been taken as an indication of 'neurogenicity', that is, a representation of the number of stem cells in a particular region, niche, or age; (ii) the presence of cells within a single neurosphere has been taken to imply clonality, that is, an indication that all cells have originated from a single stem cell; (iii) the diameter of a neurosphere has been used as an assessment of mitogenic potential of specific molecules/substances; and (iv) the composition of a neurosphere has been taken to indicate lineage commitment of a clone. Clonality is the most crucial indicator of “stemness”. In recent years, the validity of the *in vitro* neurosphere assay as a measure of clonality, multipotentiality and neurogenicity has been questioned [1]–[6]. The central premise of the neurosphere assay is that each sphere is derived from a single cell and is, therefore, clonal [1] and the final readout of the neurosphere assay is the number and size of neurospheres. This premise is in itself false as both stem cells and their transit-amplifying progenitors can form neurospheres; in fact quiescent stem cells may not be detected by the neurosphere assay [1]. Further, the assumption that a neurosphere represents clonal proliferation of a single cell is also controversial. Time–lapse imaging of neurosphere cultures have shown NSCs and neursopheres to be highly motile, dynamic structures that tend to aggregate even at low cell densities [2], [7]; these structures show intrinsic, spontaneous locomotion, propelled in part by tiny beating cellular surface processes when left untouched in incubators [2], and they frequently fuse when moved during observation by the experimenter [6], thus producing an inherent error in the neurophere assay in terms of clonality, size and number of neurospheres.

Hitherto, *in vitro* experiments on neurosphere and NSC migration and aggregation have been conducted on timescales of between 30 minutes to several hours [2], [5]. We report here results of studies that we have conducted using an entirely new approach. We make use of optical trapping (optical tweezing) to dissect out the time dynamics and mechanics of the aggregation process. Specifically, our single-cell experiments on filopodia-mediated as well as membrane-mediated cell adhesion enable us to (i) quantify the most probable value of the minimum interaction time that is required for irreversible adhesion of cells to occur, (ii) to quantify the minimum distance of approach that is required before irreversible adhesion occurs, and (iii) to measure a lower limit for the adhesive forces that are generated when NSCs adhere and aggregate. Additionally, our experiments also probe post-adhesion dynamics: oscillatory motion in filopodia-mediated cell adhesion is observed in real time. Finally, we report a differential effect of Cytochalasin-D (Cyt-D), an actin polymerization inhibitor, on filopodia-mediated but not on membrane-mediated adhesion of NSCs, although removal of calcium ions inhibits both types of adhesion.

To the best of our knowledge, optical trapping has not, hitherto, been applied to quantitative studies of NSCs or neurospheres of the type that we report here. There are a few studies in the field of neuroscience where optical traps have been used for laser-guided growth of various neural cells [8], manipulation of retinal cells in culture [9], and, in combination with a laser dissector, to ablate connections in neural networks [10]. Besides, we know of no other study that provides direct and quantitative evidence that cell adhesion dynamics proceeds on timescales of only a few seconds. Another novel facet that is revealed in our study is that the time over which individual NSCs interact with each other, or individual cells interact with a neurosphere, is the dominant factor that determines whether or not processes like adhesion and aggregation occur: a minimum time before which dynamics cannot proceed is discovered by us. We probe the temporally- and spatially-resolved dynamics of both filipodia-mediated as well as surface membrane-mediated adhesion. Quantification of these dynamics is specifically made possible in our experiments because the optical trap enables us to manipulate individual NSCs into proximity to each other and to neurospheres. The novel use of optical trapping in conjunction with fluid flow technology to study functional dynamics of NSCs has also enabled us, for the first time, to quantify a lower limit of the adhesive force by which NSCs adhere and aggregate. This finding has implications for the neurosphere assay in that our quantification of a lower limit for the adhesive forces that are generated when NSCs adhere and aggregate indicates that adhered NSCs are robust entities in that they cannot be separated by a force whose magnitude is less than a few tens of picoNewtons.

Results from *in vitro* experiments conducted on a macroscopic level may be linked to our single-cell experiments. Consider a collection of individual NSCs in a petri dish that is shaken. Each cell in the petri dish will have a finite probability of adhering to neighboring cells; there is also a finite probability that aggregated cells within the petri dish will disassemble upon shaking. Our single-cell results quantify a minimum value of force that is required for disassembly and minimum values of interaction time and cell-cell separation before adjacent cells adhere to each other.

In order to validate our experimental results demonstrating the robustness of NSC aggregates, we have also computed values of forces that may be experienced by the adhered cells using two different approaches based on a fluid dynamics model as well as a probabilistic approach that uses simple kinetic considerations. Both sets of theoretical results confirm that once aggregated, neurospheres cannot disassemble by being subjected to vigorous shaking or by thermal effects. In other words, our experimental and theoretical results indicate that the probability of aggregation is much larger than the probability of disassembly of NSCs *in vitro*. Our findings lend credence to the notion that the neurosphere assay may not be a valid measure of clonality and “stemness”.

## Materials and Methods

### Ethics statement

Animal procedures were approved by the Centre for Excellence in Basic Science's Institutional Ethics Committee (Approval ID: CBS-EC-010-02).

### Reagents

Dulbecco's modified Eagle's medium (DMEM), Neurobasal medium, Hanks balanced salt solution (HBSS), HEPES buffer, phosphate buffered saline 10x, B-27 and F-12 supplements, fetal bovine serum, Glutamax, and antibiotics were purchased from Invitrogen (NY, USA); basic fibroblast growth factor (FGF-2) was purchased from R & D Systems (USA). Percoll, all-trans retinoic acid, polyornithine, laminin, and ethylene glycol tetra-acetic acid (EGTA) were purchased from Sigma-Aldrich (USA). Antibodies mouse anti-nestin, rabbit anti-musashi, mouse anti Sox-2, rabbit anti-GFAP, mouse anti-O4, mouse anti-O1mouse, and anti-NeuN were purchased from Millipore (USA); mouse anti-BrdU and DAPI were from Sigma-Aldrich (USA). Mouse and rabbit conjugated Alexa-Fluor-488 and Alexa-Fluor-555 conjugated secondary antibodies were purchased from Invitrogen (USA). Phalloidin conjugated Alexa-Fluor-488 and Cytochalasin-D were purchased from Invitrogen (USA). All other fine chemicals were from Sigma-Aldrich (USA).

### Animal tissues

Brains dissected from young adult (p45–55 days; 250–280 gm) timed and mated Sprague Dawley male rats were procured from the registered animal facility of Reliance Life Sciences (Mumbai, India), after euthanizing according to approved guidelines. The dissected brains were transported in HBSS medium buffered with HEPES on ice for fine dissection and further processing.

### Cell culture and immunocytochemistry

The protocol for neurosphere culture was adapted from a procedure described earlier [11]. Hippocampi were dissected from brains of adult (200–250 gm) male Sprague-Dawley rats and neural progenitor cells were isolated using a Percoll density gradient centrifugation method as described elsewhere [12]. To obtain fairly pure populations of neural progenitors, the isolated cells were initially plated on poly-ornithine (20 µg/ml) and laminin (10 µg/ml)-coated T-25 flasks in DMEM/F-12 medium supplemented with B-27 and 40 ng/ml FGF-2 at 37°C in a humidified atmosphere with 5% CO_2_. After about 3 weeks, dense colonies of proliferating progenitor-like cells were manually stripped, mechanically dissociated and grown as neurospheres in uncoated 6-well plates in Neurobasal medium supplemented with B-27 at a density of 10 cells/µl, with 20 ng/ml FGF-2. Proliferating cells in the neurospheres were detected by BrdU incorporation upon overnight incubation with 40 µM BrdU followed by immunostaining. Neurospheres were differentiated by transferring them to differentiation medium (Neurobasal B-27 medium, 1% fetal calf serum, 100 ng/ml all-trans retinoic acid, 1ng/ml FGF-2) for about two weeks.

For all immunocytochemistry, neurospheres were plated onto poly-ornithine (50 µg/ml) and laminin (10 µg/ml) coated 16-well Lab-Tek chamber slides.

For characterization of neural progenitors, adherent hippocampal neurospheres plated onto coated 16-well Lab-Tek chamber slides, were fixed with 4% paraformaldehyde for 15 minutes. The following primary antibodies were used: mouse anti-nestin (1∶20), rabbit anti-musashi (1∶200), mouse anti sox-2 (1∶500), mouse anti-O4 (1∶50), followed by detection with the respective Alexa-Fluor conjugated secondary antibodies and counterstaining with DAPI (50 µg/ml). (see Fig. S1).

For BrdU immunocytochemistry, after fixation and acid hydrolysis, cells were incubated overnight with mouse anti-BrdU antibody (1∶300) and then exposed to secondary antibody (Alexa-Fluor 555-conjugated anti-mouse IgG, 1∶1000). Cells were counterstained with DAPI (50 µg/ml) and were then mounted with Fluoromount. (see Fig.).

To examine glial or neuronal differentiation of progenitors, neurospheres were incubated with primary rabbit anti-GFAP (1∶1000), anti-NeuN (1∶100) and anti-O1 (1∶100) antibodies followed by detection with respective Alexa-Fluor conjugated secondary antibodies. Neurospheres were counterstained with DAPI (50 µg/ml) and were then mounted with Fluoromount (Sigma). All imaging was performed using an epifluorescent (Nikon 90i) microscope (see Fig. S1).

For F-actin staining, dissociated neurosphere cells were stained with phalloidin conjugated to Alexa-488. Briefly, cells were fixed 0.25% glutaraldehyde and permeabilised with 0.05% Triton X-100. After blocking with 3% bovine serum albumin, cells were incubated with Alexa-Fluor 488-tagged phalloidin, washed and mounted for confocal imaging with a Carl Zeiss LSM 5 Exciter confocal microscope. Several optical slices of each cell were recorded at a distance of 0.5 µm in between two slices. All such slices were subsequently overlaid and averaged to obtain the final image. 3-D images were constructed from slices using *Image J* software.

### Optical trap and flow cell

**Figure 1:**
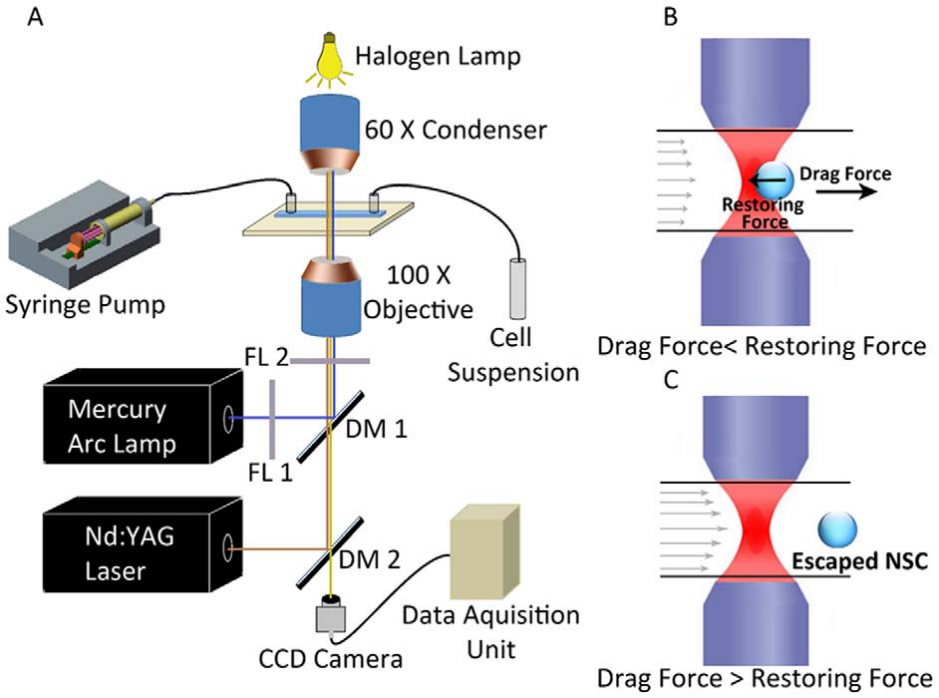
The experimental set-up and its principle of operation. A) Schematic diagram of the optical trap apparatus; B) Sketches depicting a neural progenitor trapped within the flow cell; and C) the principle of escape force determination.

Our experimental set-up comprised optical tweezers coupled to a liquid flow cell (see [13]–[17] for details of the apparatus, and a schematic diagram shown in Fig. 1A). In brief, it consists of a 1 W Nd:YVO_4_ laser (Photop Suwtech, DPIR-2500) with a 2-mm beam that was expanded to 8 mm. This beam was coupled to an inverted microscope (Nikon TE 2000U). Optical trapping was achieved by focusing 1064 nm wavelength, linearly-polarized light from the laser through a 100X oil-immersed objective (numerical aperture 1.3) onto a flow cell. The diameter of the focused laser spot (typically measured to be in the range 0.6–1 µm) was much smaller than cell diameters (typically 6–8 µm). The laser-cell interaction volume is, consequently, very small and is located at or near the center of the cell, well away from the periphery where processes leading to adhesion are expected to occur. The trapped cells were imaged through the same objective by a CCD camera coupled to a computer for real time recording; individual movie frames were analyzed with *Image J* software. Our flow cell was of rectangular cross section (100 μm height, 8 mm width) and was 48 mm long. It was constructed using glass slides and was connected to a peristaltic pump (Pharmacia LKB-Pump P-1) which was used to maintain continuous flow of the cell suspension through the glass chamber at constant (and controllable) flow speeds that ranged from 5 µm s^−1^ to 150 µm s^−1^. Flow speeds were measured in a separate experiment by imaging flowing 2 μm polystyrene beads and monitoring them in a series of sequential frames (separated by 40 ms) of a real-time movie. The laser power at the trap was directly measured by an integrating sphere attached to a calibrated photodiode. Our experiments were conducted over a range of powers, from 5 mW to 50 mW. At power levels less than 5 mW trapping was negligible while power levels in excess of 50 mW were avoided so as to minimize the possibility of inducing cell damage [13]–[19].

### Trapping procedure

Neurospheres were mechanically dissociated by trituration to obtain single neural progenitor cells or smaller neurospheres. These were suspended at a density of 10^3^ cells/µl in 1x PBS. 10 µl of this sample was loaded onto an agarose-coated coverslip and placed in the optical trap.

Within the trap, a single cell was randomly selected from within the microscope field of view, was trapped at the focal volume, and brought in close proximity to other cells or neurospheres. To determine the minimum adhesion time, the cells were kept close to each other (such that the cells came in contact with each other either through their membranes or filopodia) for increasing amounts of time and then the trapped cell was pulled away so as to determine whether adhesion had occurred or not. The minimum time required for the cells to irreversibly adhere to each other, determined by analyzing individual frames from real-time movies, was designated as the minimum time needed for adhesion. A large number of cells (typically in excess of 50 per measurement) were studied and a histogram was plotted so as to yield a distribution of values of minimum time for irreversible adhesion. The temporal information was obtained over a range of values of trap strength (laser power); the measured histograms were found to be qualitatively independent of laser power over the range of values used in the present series of experiments. Furthermore, as the time information was obtained on the basis of imaging from real-time movies, the value of minimum time was essentially independent of the geometry of the trapped cells, namely on whether it was an individual spherical cell in the trapping volume or two spherical cells joined together.

Two individual cells or neurospheres were said to be adhered under the following two circumstances. (i) In case of cell-cell adhesion, if upon moving the trapped cell, the adhered cell also moved (with no relative velocity between them) the cells were said to be adhered. Also, if the untrapped cell among the two adhered cells was attached to the glass coverslip, and on moving the trap focus, the trapped cell did not move, but remain attached to the other cell the cells were considered, adhered. (ii) In case of cell-neurosphere adhesion, a similar assay was followed. The cell and the neurosphere were considered to be adhered if, upon moving the trap focus, the trapped cell did not move along with the trap but remained attached to the neurosphere.

The adhesion strength was deduced by measuring the maximum force exerted by the optical trap without joined cells (or neurospheres) being induced to separate; we take this to be the lower limit of the adhesion force. The trapping force on a single NSC was determined by the conventional escape velocity method [17]. The single cells were trapped in a flow cell and liquid medium was allowed to flow around it at progressively increasing velocities. As the medium flows, it provides a drag force on the cell. At low flow speeds, this drag force is counteracted by a restoring force from the optical trap (Fig. 1B). As the flow velocity increases, the drag force increases. Finally, when the drag force becomes more than the maximum restoring force that he trap can apply, the cell escapes the trap and flows away (Fig. 1C). Thus, the measure of the drag force when the cell escapes provides a measure of the maximum trapping force. To measure the drag force, the escape velocity of the cell is calculated by analysis of the video frames just after the escape event. Then the force *F* can be denoted by 6π*ηav,* where, *v* is escape velocity, *a* is the radius of the NSC, and *η* is the medium viscosity.

For the measurements of minimum distances between the cells to be adhered, two cells, randomly chosen, were brought to close proximity and allowed to stand for the minimum adhesion time period, after which the cells were moved apart. Such trials were repeated many times with progressively shorter distances between the cells, until the cells failed to separate from each other after the minimum attachment time period. This distance between the cells, was then designated as the minimum interaction distance.

To abrogate Ca^2+^-dependent adhesion receptor pathways by depleting calcium ions, NSCs/neurospheres were treated with 1 µM, 500 µM, and 1 mM EGTA, a calcium ion chelator, for 1 hour. Such cells were then probed for membrane and filopodial adhesion through trapping.

Cyt-D provides a well-established convenient way for perturbing the actin cytoskeletal pathway [20]. Single NSCs were treated with different concentrations of Cyt-D (1 µM and 0.1 µM) for 5 minutes before fixing or trapping, to look at the effects on F-actin and cell-cell adhesion.

## Results

**Figure 2:**
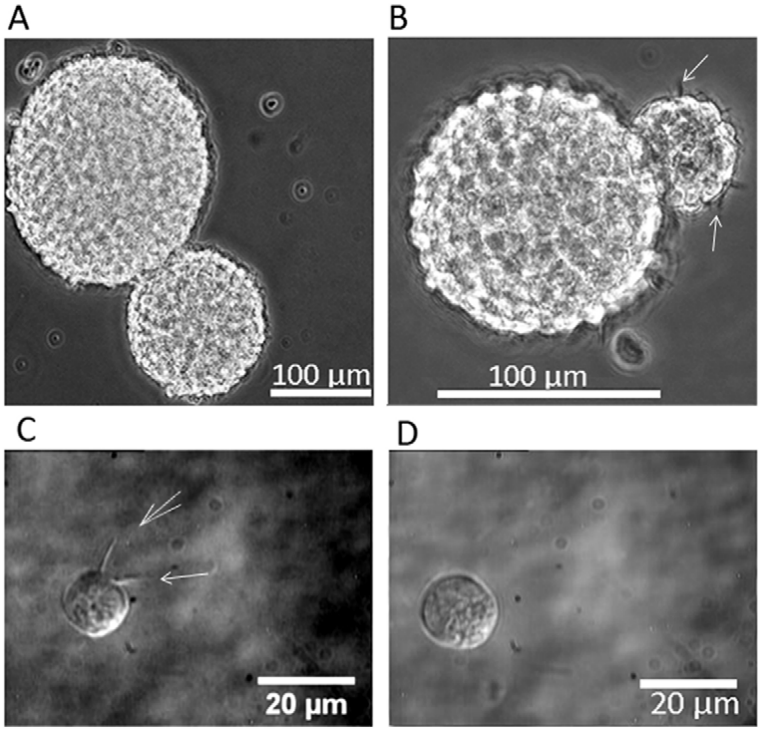
Phase contrast images of neurospheres and single neural progenitor cells from dissociated neurospheres. A) Two fused neurospheres; B) Fused neurospheres with filopodia visible on the surface; C) Differential interference contrast image of a single neural progenitor cell with filopodia; and D) a single neural progenitor cell without filopodia. Filopodia are indicated by white arrows.

Single cells in suspension formed neurospheres. Each well of the 6-well plate had, on average, approximately 60 neurospheres that tended to accumulate at each well's center. Neurospheres of varying sizes were seen to coalesce and fuse, typical images of which are depicted in Fig. 2A and B. Many of such neurospheres were seen to exhibit long and short filopodia at their surface (Fig. 2B). Upon dissociation of neurospheres, a mix of single NSCs were obtained (Fig. 2C, D), some of which had prominent filopodia, both short and long (Fig. 2C).

**Figure 3:**
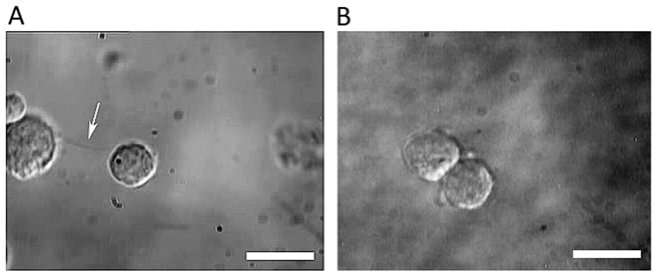
Adhesion of neural progenitor/stem cells (NSCs). A) NSC adhesion mediated by a long filopodial bridge (indicated by the white arrow); B) Surface adhesion of two neural progenitor cells. The scale bars denote 10 µm.

By optically trapping a single NSC, we could bring it in proximity to adjacent cells or neurospheres. We observed that cell-cell adhesion or cell-neurosphere adhesion occurred spontaneously through either filopodial interaction or via surface membranes. Typical images of cell-cell adhesion of both types are depicted in Fig. 3. To probe the temporal dynamics of these processes the interaction time between NSCs was varied and it was observed that, in all instances, a most probable minimum interaction time of ∼5 s was necessary for irreversible adhesion to occur.

**Figure 4:**
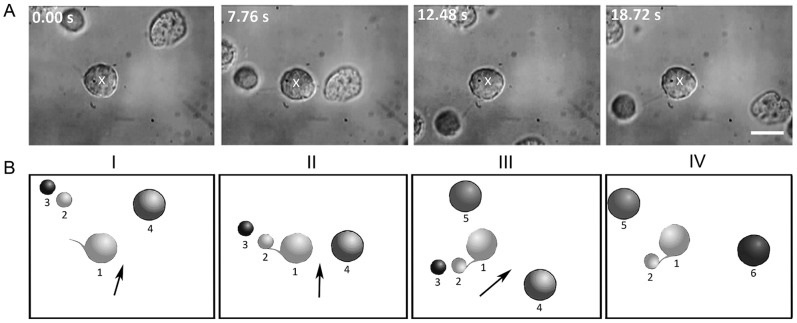
Time lapse images of cell adhesion dynamics. A) Real-time images and B) corresponding cartoon depictions showing filopodia-mediated adhesion. The white cross in image I denotes the position of the optically-trapped neural progenitor cell 1 that is made to move in the direction indicated by the arrow. Image II shows the filopodia of cell 1 coming into contact with cell 2. Upon moving cell 1 after a period of approximately 5 seconds (Image III), cell 2 also moves along, thereby indicating adhesion of cells 1 and 2, with a filopodial bridge between the two cells. Image IV (6 seconds later) shows that the cells remain adhered. The scale bar denotes 10 µm. See the real-time movie (Movie S1).

Figure 4 shows frames from a real-time movie depicting cell-cell adhesion through filopodia. (See Movie S1) The white cross in image I denotes the position of the optically-trapped NSC (marked 1 in the cartoon B). This cell was made to move in the direction indicated by the arrow. Image II shows the filopodia of the trapped cell coming into contact with an untrapped cell (marked 2 in the cartoon B). If the filopodia of the trapped cell remains in contact with cell 2 for at least ∼5 seconds, irreversible adhesion takes place. This is indicated by cell 2 also moving along with the trapped cell (images III and IV and the accompanying cartoons). The filopodial bridge between the two cells is clearly visible in panels II, III, and IV of Fig. 4. Image IV was taken 6 seconds after the initial adhesion event and demonstrates that the adhesion persists.

**Figure 5:**
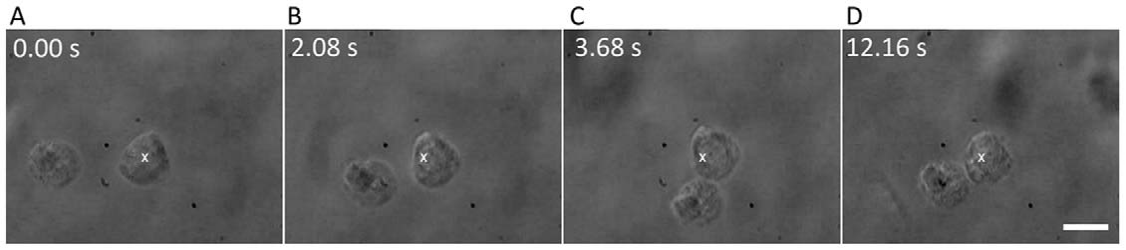
Time-lapse images of membrane-mediated cell adhesion dynamics. A) – C) Time lapse images from a real-time movie (Movie S2) showing surface adhesion of two neural progenitor cells without filopodia. The white cross denotes the position of the optically-trapped cell being brought towards the second cell. Irreversible adhesion occurs after the two cells are in contact by their surface membranes for about 5 seconds. The scale bar denotes 10 µm.

We also studied cell-cell adhesion that proceeds via membrane interactions and some typical images from a real-time movie are depicted in Fig. 5 (see Movie S2). As in the case of images in Fig. 4, the white cross denotes the position of the optically-trapped NSC being brought towards another. As before, irreversible adhesion occurs after the two cells are in contact for at least ∼5 seconds.

**Figure 6:**
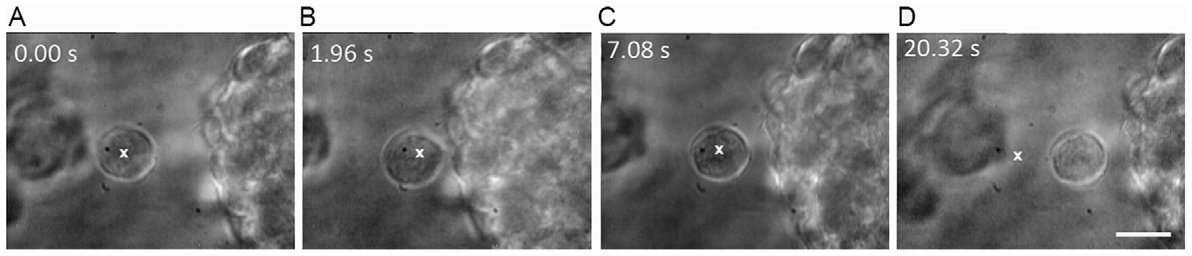
Time-lapse images of filopodia-mediated cell adhesion dynamics. A) – D) Time lapse images from a real-time movie (Movie S3) showing adhesion of a neural progenitor cell to a neurosphere via filopodial interaction. The white cross denotes the position of the optically-trapped cell being brought towards the neurosphere. The single cell adheres to the neurosphere after approximately 5 seconds. Note the faintly visible filopodial bridge connecting the neural progenitor to the neurosphere in panel C). The scale bar denotes 10 µm.

The dynamics that occur when a trapped NSC is brought close to a neurosphere are similar, and are depicted as frames from real-time movie in Fig. 6 (see Movie S3). As before, the white cross denotes the position of the optically-trapped NSC. Irreversible adhesion occurs after the NSC is in contact with the neurospheres for approximately 5 seconds. This is demonstrated by moving the trap away (position indicated by the white cross in panel D in Fig. 6) and observing that the NSC remains adhered to the neurosphere. Note that in panel D of the figure the NSC is not conjoined to the surface of the neurosphere, indicating that adhesion occurs via a filopodial interaction even though the filopodia are barely resolved with our optical microscopy.

**Figure 7:**
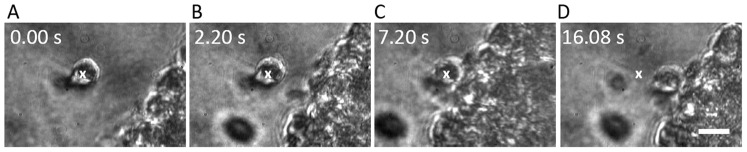
Time-lapse images of membrane-mediated cell adhesion dynamics. A) – D) Real-time images (Movie S4) demonstrating adhesion of a single progenitor cell to a neurosphere in the absence of filopodia. The white cross denotes the position of the optically-trapped cell being brought towards the neurosphere. Panels C) and D) show that irreversible adhesion occurs after approximately 5 seconds. Note that the surfaces of both are juxtaposed closely, indicating membrane-membrane adhesion. The scale bar denotes 10 µm.

Adhesion of a NSC to a neurosphere occurring via membrane interactions in the absence of filopodia was also observed (Fig. 7) (Movie S4). As in the case of filopodia-mediated adhesion discussed above, a minimum interaction time of ∼5 s was found to be necessary for irreversible adhesion to occur. Panel D in Fig. 7 shows that 9 seconds after the initial contact is made between the initially optically trapped NSC and the surface of the neurosphere, the adhesion persists: moving the position of the optical trap (marked X in the figure) does not result in movement of the NSC away from the neurospheres surface.

**Figure 8:**
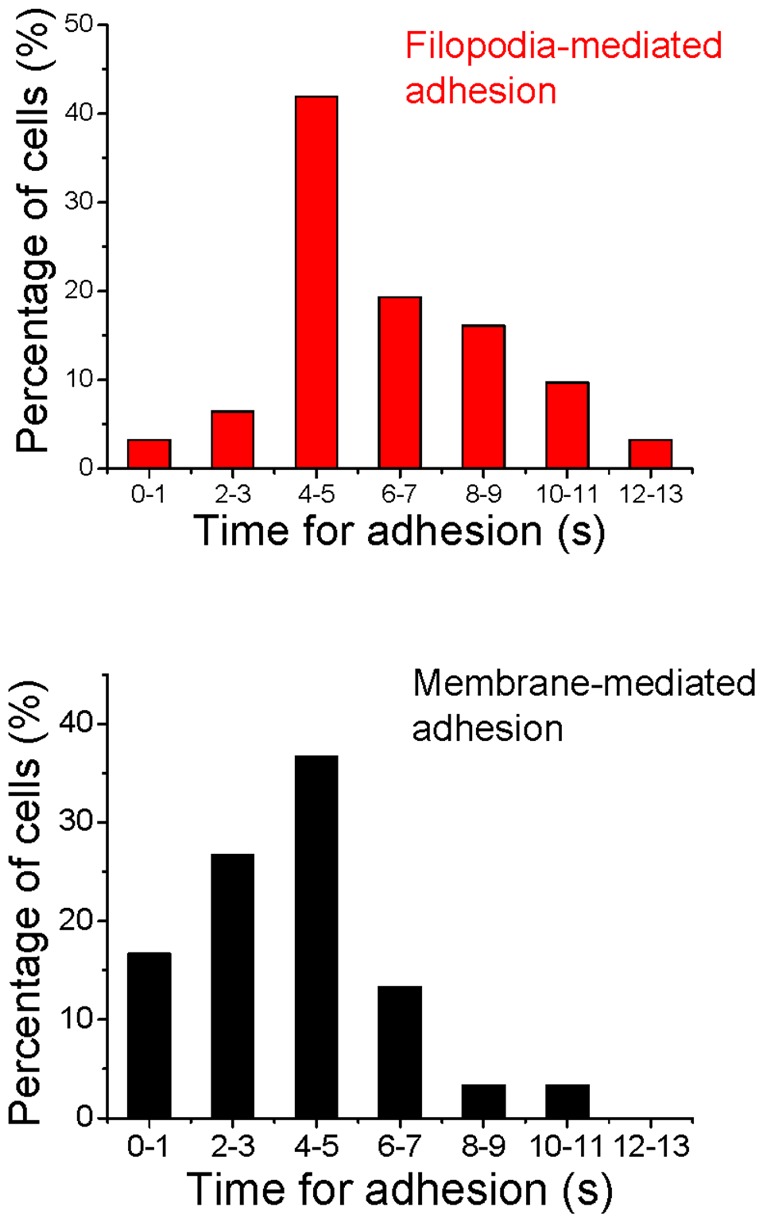
Histograms showing the percentage of cells that underwent irreversible adhesion as a function of contact time for filopodia-mediated adhesion (top panel) and membrane-mediated adhesion (lower panel). A minimum of 50 cells were used for each measurement. Time determination was by analyzing individual frames from real-time movies, with each frame being temporally separated from adjacent frames by 40 ms.

In our discussion up till now we have referred to the minimum time required before irreversible adhesion to be ∼5 s. This value pertains to the most probable value of the minimum time. Measurements of minimum time, conducted by us on a large number of cells (for each such measurement, 50 cells or more were examined), reveal a distribution of values of minimum time for irreversible adhesion via both filopodial as well as membrane interactions. Data are depicted in Fig. 8 in the form of histograms and shows a range from time periods as short as 1 s to as long as 12–13 s, with the most probable value of ∼5 s being applicable to both filopodia-mediated and membrane-mediated adhesion. Time determinations were accomplished by analyzing frames of real-time movies; subsequent frames were temporally separated by 40 ms, and this provided the least count for our time measurements.

**Figure 9:**
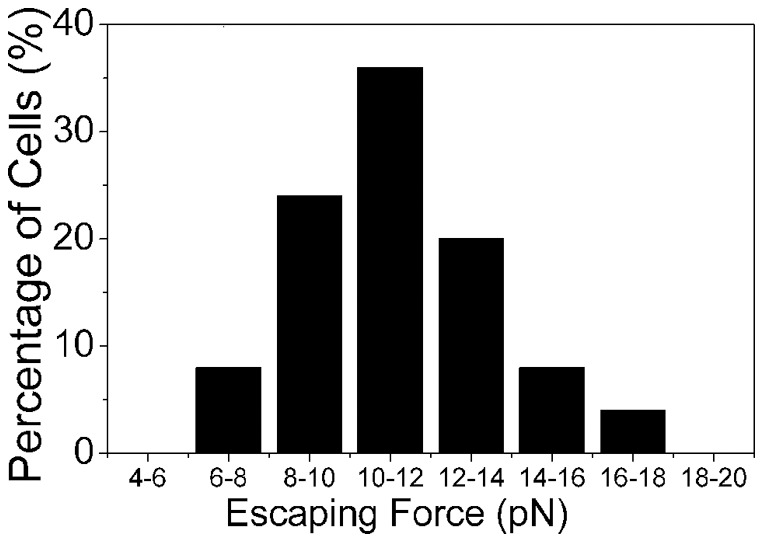
Histogram depicting the lower limit of the force required to separate two adhered neural progenitor cells. This corresponds to the force required by a cell to escape from the flow cell of the optical trap schematically depicted in Fig 1B and 1C. Data presented pertains to measurements made on 56 cells.

What are the magnitudes of the forces that result in irreversible adhesion that has been observed in Figs. 2,3,4,5,6,7. The use of the optical trap permits us to evaluate at least a lower limit for such adhesive forces. The method we employ for these measurements has been described in relation to our description of our experimental procedures (Fig. 1). Figure 9 shows a histogram of force required by a single NSC to escape from the focal volume of our optical trap. This escape force represents the lower limit for the adhesive force and spans the range from about 6 pN to as much as 18 pN, with a most probable value of 10–12 pN. We note that the value of adhesive force that we deduce using the escape force method has an in-built inaccuracy. The methodology is applicable to spherical objects that are optically trapped, and our trapped NSCs are not perfect spheres. However, since the escaping force and the radius of the escaping sphere are linearly dependent on each other, and the difference between the long and short axes of our trapped NSCs never exceeds 10% of the long axis, the error in the escaping force value that we deduce is readily estimated to be within 10%.

**Figure 10:**
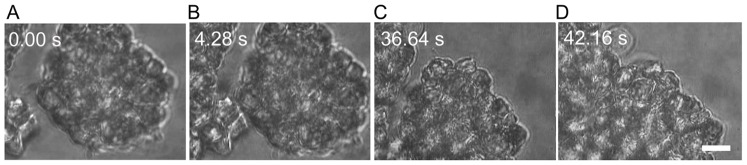
Time lapse images from a real-time movie (Movie S5) showing adhesion and subsequent fusion of two neurospheres. A) – C) Adhesion occurs when the two neurospheres are brought into close proximity by the optical trap for approximately 5 seconds. D) The two neurospheres fuse completely after about 40 seconds. The scale bar denotes 10 µm.

**Figure 11:**
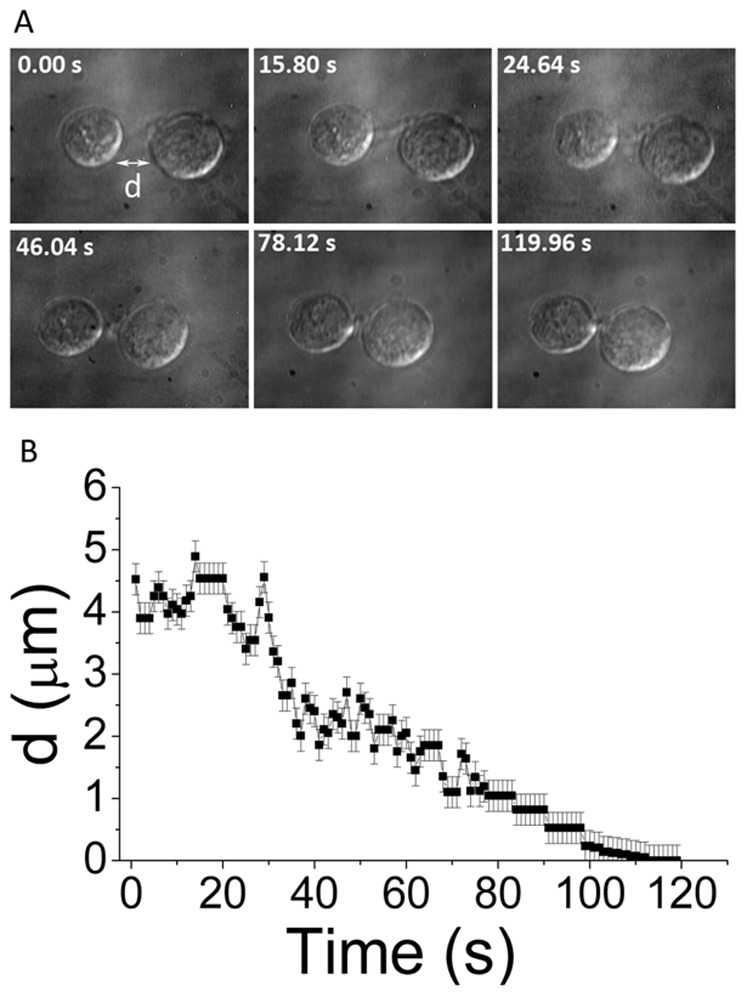
Temporal events post filopodia-mediated adhesion of two neural progenitors. A) depicts time lapse images from a real time movie and B) shows a graphic quantitation. 'd' is the minimum distance between the surfaces of the two neural progenitors. The scale bar denotes 10 µm.

Figure 10 shows the sequence of events that leads to the adhesion of two neurospheres. The optical trap is used to bring the two neurospheres into close proximity (panel B). After a further period of ∼38 s complete fusion of the two neurospheres is observed (panel D). The real-time movie clip from these time-lapse images have been extracted (Movie S5) shows the presence of several filopodia on the neurosphere surfaces which appear to mediate the adhesion process. Note the apparently slow movement of the two neurospheres towards each other, eventually leading to complete fusion. In order to probe the temporal dynamics in quantitative fashion, experiments were conducted on two single NSCs, one of which had filopodia that mediated cell-cell adhesion. Figure 11A shows the time evolution of the post-adhesion events whereby filopodial contractions bring the two NSCs into juxtaposition. Quantification of such post-adhesion events is graphically depicted in Fig. 11B where *d* denotes the minimum distance between the surfaces of the two NSCs. Note that for the first 20 s, there are oscillations but the mean value of *d* remains essentially invariant. Thereafter, there is a steady decrease in the value of *d* while small-amplitude oscillations seem to persist.

**Figure 12:**
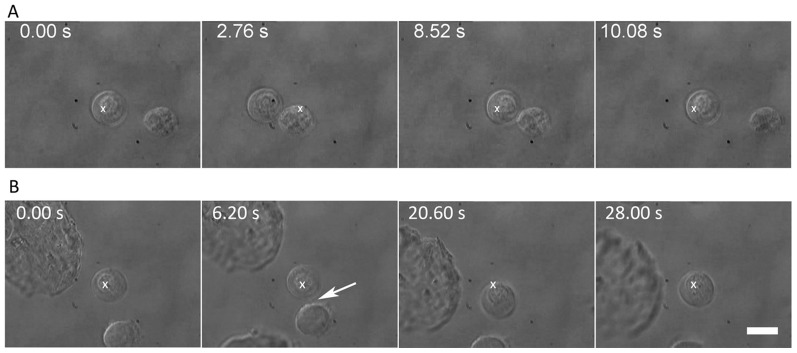
Time-lapse images from real-time movies depicting the outcome of a 1-hour treatment of NSCs with EGTA, a calcium ion chelator. A) absence of membrane-mediated adhesion between the two EGTA-treated NSCs brought into close contact for ∼6 s (data extracted from Movie S6); B) filopodial contact between the two EGTA-treated NSCs results in adhesion at ∼5 s. Subsequently, the NSCs remain adhered when the trap focus is moved slightly, but they are eventually pulled apart ∼8 s later. The scale bar denotes 10 µm.

Cell-cell adhesion, whether membrane- or filopodia-mediated, may occur as a result of Ca^2+^-dependent, or Ca^2+^-independent cell adhesion molecule (CAM) interactions and signaling. In neural cells, examples of the former are the cadherins and integrins, and of the latter are the NCAMs. Figure 12 depicts as time-lapse images from real-time movies the outcome of a 1-hour treatment of NSCs with EGTA, a calcium ion chelator on (A) membrane-mediated and (B) filopodial-mediated cell-cell adhesion. Figure 12A (extracted from Movie S6) shows absence of membrane-mediated adhesion between the two EGTA-treated NSCs that are brought into each other's proximity for a period of ∼6 s. Figure 12B shows that filopodial contact between the two EGTA-treated NSCs results in adhesion at ∼5 s and these NSCs remain adhered when the trap focus is moved slightly. Eventually they are pulled apart ∼8 s later. Further work is clearly necessary in order to quantify the strength of adhesion.

**Figure 13:**
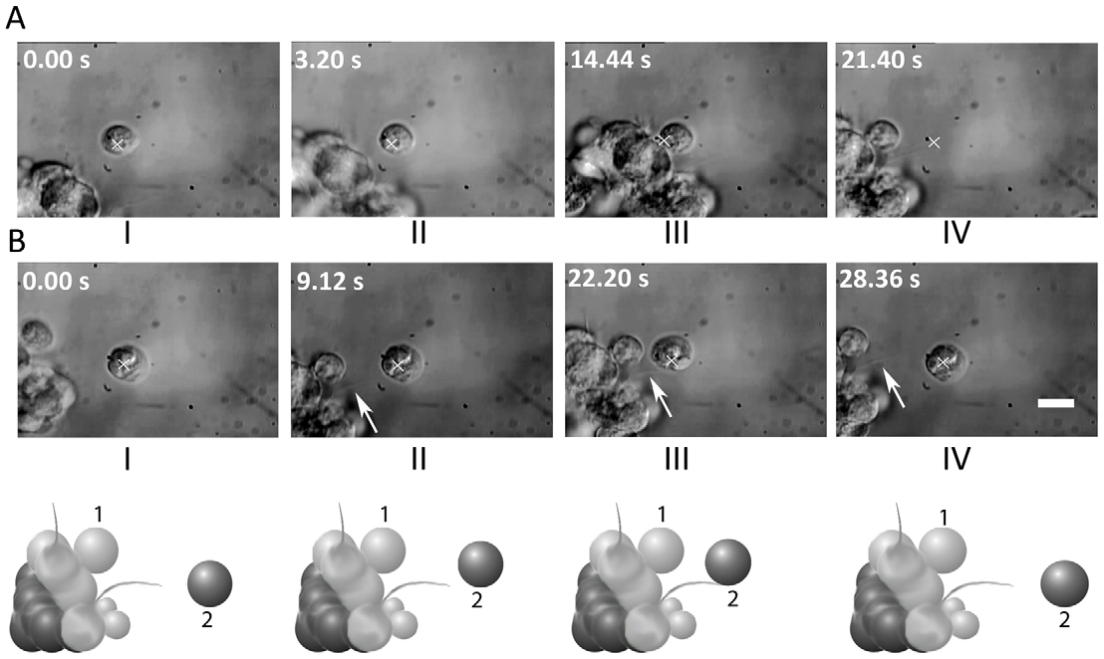
Differential effect of 0.1 µM Cytochalasin-D treatment on membrane and filopodial adhesion. A) Time-lapse images from a real-time movie (Movie S7) showing a cell (initially at the trap focus, X) approaching a neurosphere. As the trap is moved away (panel IV), the cell is seen to remain adhered to the neurosphere. B) Time-lapse images from a real-time movie (Movie S8) of a single cell approaching a filopodium on the neurosphere. Despite close proximity (for up to 7 seconds), no filopodial adhesion takes place: the cell remains within the trap focal volume, X, as it is moved away from the neurosphere. The cartoon below panel B is a chimera depicting both phenomena (as cells numbered 1 and 2, respectively). The scale bar denotes 10 µm.

The role of filopodia in the adhesion process has also been studied by treating NSCs with Cytochalasin-D (Cyt-D), an inhibitor of actin polymerization at a concentration of 0.1 µM. A 1 µM concentration of Cyt-D was found to be cytotoxic. Figure 13A depicts time-lapse images of a Cyt-D (0.1 µM)-treated NSC being made to approach a neurosphere: membrane-mediated adhesion is seen to occur (depicted as cell 1 in the accompanying cartoon; see also the Movie S7). In contrast, Fig. 13B shows that filopodia-mediated adhesion simply does not occur after Cyt-D treatment (depicted as cell 2 in the accompanying cartoon; see also Movie S8). Note that the trapped NSC rotates counterclockwise upon briefly attaching to the neurosphere by the filopodium. The propensity of filopodia in hippocampal neurite growth cones to rotate counterclockwise around the longitudinal axis due to the involvement of myosin V has been noted before [21].

**Figure 14:**
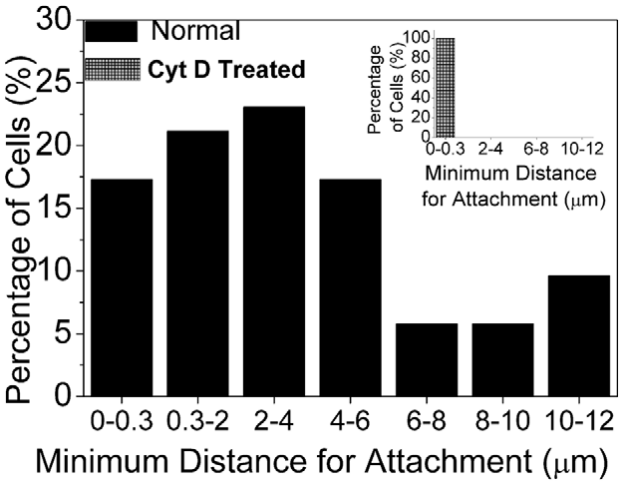
Histogram depicting the minimum distance of approach before two cells adhere to each other through filopodial interactions. Inset shows the corresponding histogram when the cells have been treated with 0.1 µM Cytochalasin-D.

The most probable minimum approach distance that is required for irreversible adhesion to occur has been quantified for untreated NSCs and those that are Cyt-D treated, and the results are shown in Fig. 14. Most of the cells exhibit a high propensity for attaching to each other over minimum approach distances of up to 4–6 µm. However, the histogram clearly brings out the fact that long-range (as long as 10–12 µm) adhesion also occurs which can only be filopodia-mediated. However, after Cyt-D treatment, the dramatic observation is that no such long-range adhesion occurs (see inset to Fig. 14), indicating that only membrane adhesion occurs requiring minimum approach distances of less than 0.3 µm.

**Figure 15:**
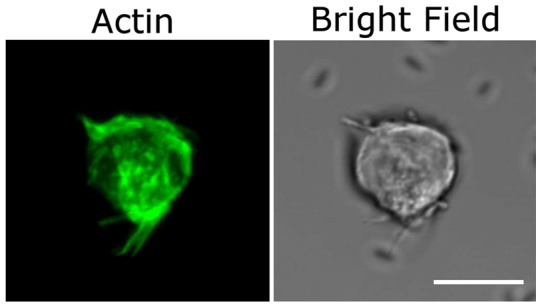
Confocal and DIC images of F-actin staining of a normal neural progenitor cell. Note the presence of F-actin in all filopodial projections as well as in the cytoskeleton of the cell. Scale bars represent 10 µm. (See Movie S9 for 3D reconstruction).

**Figure 16:**
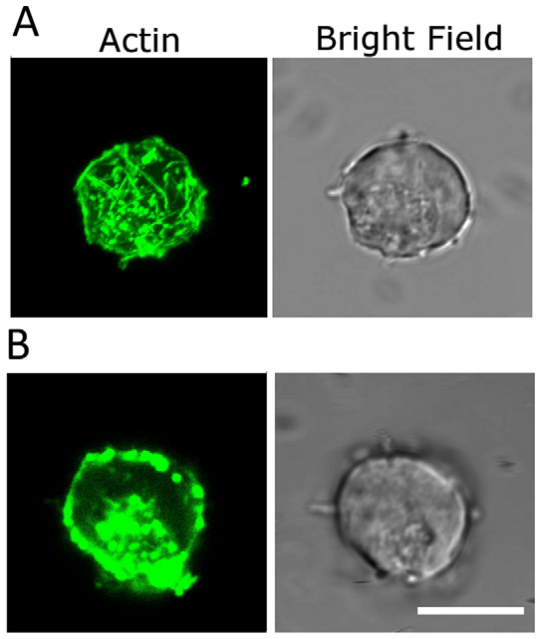
Typical confocal and DIC images of F-actin staining of Cytochalasin – D treated neural progenitor cells. Note the absence of actin staining in the filopodial projections of the neural progenitor in A) even though there are prominent F-actin filaments visible within the cell body (See Movie S10 for a 3D reconstruction). The neural progenitor in B) shows not only an absence of F-actin staining in the filopodial projections but a significant disruption of cytoskeletal F-actin as well. Scale bars denote 10 µm.

The morphologies of normal and Cyt-D treated NSCs stained for F-actin are imaged in Figs. 15 and 16. This is representative of a total of ∼20 NSCs that were studied in each case. Panel A shows F-actin staining within the cytoskeleton and filopodia of a single NSC. A 3-D rendition of the F-actin structures is shown in a movie clip (Movie S9). Cyt-D treatment disrupts both cytoskeletal and filopodial F-actin in varying degrees, and this is depicted in panels A and B in Fig. 16 (Movie S10). Note that the bright field images show distinct short filopodia whereas the corresponding phalloidin-stained images do not yield any evidence of F-actin within these membranous filopodial protrusions. The absence of long filopodia in these images is a consequence of the cell fixation procedure which seems to degrade the longer filopodia into shorter structures. It is pertinent to note that simultaneous actin staining and testing for adhesion was not possible. In our experiments, NSCs were treated with Cyt-D and tested for adhesion in the optical trap. From the same sample, an aliquot was taken and stained for actin, which revealed variable actin disruption. In the course of our experiments on many (>50) cells no correlation was established between the repeatability of adhesion dynamics and the variability in the appearance of the actin-stained cells: the range of values of minimum time for irreversible adhesion remained essentially unchanged.

## Discussion

There is considerable contemporary interest in the neurosphere assay [1]–[7], with several protagonists who are strongly in favor of this assay and others who express reservations about the validity of this assay as a measure of clonality [2], [4], [5] and as a predictor of the *in vivo* number of stem cells in a tissue [22]. In order to explore this area of contemporary interest in greater depth we have adopted an entirely new technique that enables fresh insights to be generated. We choose to employ an optical trapping method to which is coupled a flow cell that enables us to quantitatively explore the spatio-temporal dynamics of cell-cell and cell-neurosphere adhesion. Our new optical technique enables hitherto unavailable information to be obtained on the most probable minimum time and minimum distance of approach required for irreversible adhesion of proximate cells to occur. Our experiments also allow us to study and quantify the spatial characteristics of filopodial- and membrane-mediated adhesion, and to probe the functional dynamics of NSCs to quantify a lower limit of the adhesive force by which NSCs aggregate. Furthermore, we have also explored the effect of the removal of calcium ions, as well as F-actin disruption on the dynamics of such adhesion events.

In earlier studies [2] continuous time-lapse video microscopy was used to show that neurospheres were drawn towards each other on time scales of 30 minutes to 3 hours, resulting in spontaneous fusion of two neurospheres. Such fusion occurred without external intervention, and relied on filopodia-driven locomotion. Such fusions were shown to occur independently of culture conditions such as plating density or whether the neursopheres were primary or passaged, as also of tissue, age, species, and site of origin [1]. Neurosphere assays should ideally be performed at clonal densities, although, this number varies over a very wide range, from 0.2–20 cells/µl, in different studies [1]. True clonality can only be ensured by plating single cells in microwells. Cell density, however, has a direct influence on cell growth and proliferation due to release of growth factors in the medium, and sphere formation is markedly reduced when cells are plated singly [1]. Neurospheres have been observed to aggregate and fuse not only at high seeding cell densities, but at low densities too [2], [7]. The rate of cellular proliferation alone cannot account for the eventual size attained by the neurosphere [2]. Additionally, single floating cells have also been observed to fuse [2]. In another work [6] it was pointed out that movement-induced aggregation readily occurs even upon the slightest amount of disturbance experienced by neurospheres during the culturing process. Migration rates of cells and neurospheres have also been quantified by means of time-lapse microscopy [5], and typical speeds have been measured to be in the range 5–10 µm/h. These values imply that the force (of the order of tens of femtoNewtons) that such cells can intrinsically generate (when in an *in-vivo* condition, as in a culture dish) is about five orders of magnitude less than the maximum trapping force generated in our experiments. This provides quantitative justification for our observation that when such cells are adhered they cannot be separated by a force less than 18 pN, a value that is far in excess of what may be generated if culture dishes with cells are physically moved or shaken (as in the process of observation). In order to estimate shearing forces that may be experienced by the adhered cells upon such movement we have adopted two different approaches involving fluid dynamic calculations as well as probabilistic calculations based on simple kinetic considerations. Both sets of calculations pertain to an adhered pair of cells.

The fluid dynamic approach assumes the culture dish to be rectangular in shape, with infinite length. This allows us to carry out a simpler one-dimensional computation of an upper limit of forces that are generated upon movement of such a dish (for details of our computational method, see Text S1). For 35 mm and 90 mm plates, our computations reveal that shearing forces of between 0 and 10 pN are experienced by cells within ∼2 mm of the center of the plate. In the case of 6-well plates, shearing forces of between 0 and 10 pN are experienced by cells located within 0.2 mm of the center. These numbers indicate that once NSCs are adhered to each other, their disassembly cannot occur upon simple shaking. Our one-dimensional analysis are of relevance in the center portion of the culture dishes; near the edges the one-dimensional model breaks down because of the curvature of fluid flow lines in real culture dishes. This one-dimensional treatment is relevant to most experimental situations as evidence exists that free-floating cells have a higher tendency to coalesce in the central portion of culture dishes [2]. We note that our numbers for shearing force pertain to the following situation: (i) the plates are vigorously shaken at a frequency of 0.5 Hz and (ii) the maximum extent of to-and-fro shaking motion is ∼10 mm. In real situations, culture dishes of neurospheres that are inadvertently shaken in the course of observations and manipulations experience considerably lower frequencies and amplitudes. This implies that the real forces will be much smaller. In that sense, our shearing force values represent upper limits.

We also adopted a simple kinetic approach to estimating separation forces (for details, see text S1). These calculations are relevant to culture dishes of any shape, and the cells may lie anywhere within the dish. The relevant parameter here is the temperature of the fluid in which the cells are suspended. The motion of the cell due to Brownian energy is assumed to result in a straight line trajectory (ballistic motion) at a mean velocity that is sufficient to overcome the Stokes drag force. The shortcoming of this approach is that a cellular mass needs to be quantified. In the absence of information in the literature on the mass of a neural stem cell, we have taken the mass of a red blood cell as a guide. Our computations reveal that at fluid temperatures of 37 C, very small forces are generated, of the order of 10^−6^ pN. These numbers are based upon a separation time of 1 s and an inter-cellular adhesion distance of 10 nm. The former pertains to the time taken for one bond between two cells to break [23]; the latter number is consistent with atomic force microscopy and cryo-electron microscopy data on cadherin interactions [24], [25]. The results of our kinetic approach also indicate that once NSCs are adhered to each other their disassembly cannot occur through thermal agitation.

It is pertinent to note that in the case of cell-neurosphere or neurosphere-neurosphere aggregation, the Brownian forces as well as fluid dynamic forces are expected to be much less than those that we have discussed above in connection with cell-cell adhesion.

These findings have important implications for the neurosphere assay: neurosphere formation by cell aggregation is a robust process that cannot be readily disrupted without the use of external forces whose magnitudes are larger than a few tens of picoNewtons. Our results also show that, once formed, neurospheres can also fuse to other neurospheres, giving rise to larger fused entities. These neurosphere-neurosphere fusions also require a most probable minimum time of ∼5 s for irreversible adhesion to occur. After this interaction time the larger fused entity also appears to be extremely robust. As already noted, our measurements on a large number of cells (at least 50 cells per measurement) reveal that the value of ∼5 s for minimum time before irreversible adhesion occurs represents the most probable value. The range of time values measured by us was from 1 s to ∼12–13 s, for both membrane-mediated and filopodia-mediated adhesion. The temporal distribution remained qualitatively invariant over the range of laser powers (thereby, trap strengths) used in our experiments.

Our present observations are in excellent qualitative accord with time-lapse microscopy results [5]. However, the measurement technique used in the present study enables different quantitative insights to be developed into the fusion and aggregation dynamics at the cellular level. As already noted, there is a minimum cell-cell and cell-neurosphere interaction time (of about 5 s) that is necessary for irreversible adhesion to take place. It is clearly of interest to further explore this temporal facet of the dynamics by considering the role played by filopodial interactions as two cells fuse. Our measurements indicate that the 5 s minimum time applies to cell-cell fusion whether it is filopodia-mediated or not: even in the case of two cells being made to translate such that their surfaces touched each other, the same minimum time was required for membrane-mediated adhesion to occur. Although there is a paucity of information in the literature, it appears that there is little or no correlation between the number of filopodia and the size of the cell. Lobo *et al*. [26] in their study on the characterization of neurospheres describe the neurosphere cells as having many pseudopodia-like or cilium-like structures having variable length and width, although they do not specify whether these were found on large or small cells. Mori *et al*. [5] using F-actin staining of NSCs from neurospheres described mini-podia and long-podia on these cells. On the basis of the present data we have no grounds to postulate that larger cells possess more filopodia and, consequently, generate larger adhesive forces than smaller cells.

Filopodia besides serving as sensors that probe the local environment, are essential for many cell biological functions such as adhesion site formation and force generation [27]. Their role in cell-cell contact and adhesion is well established in neurite outgrowth and formation of synaptic junctions in developing neurons [28] as also in epithelial cell adhesion and during dorsal closure in Drosophila [29], [30]. In developing neurons, filopodia have been observed to attach by tip-to-tip contact, even bending by as much as 90° for this orientation and could push/pull or stretch tightly while adhered [28]. In our study, quantification of the minimum distance of attachment, an important parameter that would influence adhesion of NSCs/neurospheres *in vitro*, showed that a significant number of NSCs attached through filopodia of length greater than 6 µm, extending up to 12 µm. This is concurrent with the findings of Husainy *et al.* [31] who compiled thousands of filopodial length measurements in their fibroblast cell line. According to them such filopodial elongation could occur by loss of capping protein function or active G-actin transport within filopdia.

An advantage of our experimental technique is the ability to also monitor post-adhesion dynamics. It has been observed in our experiments that periodic filopodial contraction can also occur, resulting in the distance between adhered cells fluctuating with the lengths of the filopodia oscillating. Similar intense vibratory movements have been described in adhered filopodia of developing retinal neurons [28] and are in consonance with two recent observations that filopodia undergo repeated elongation, retraction, stabilization and persistence [27], [31]. These periodic cycles of oscillatory motion in filopodial-mediated cell adhesion could be due to actin cross-linking by fascin being replaced by myosin and α-actinin to form contractile fibers [27], or as proposed by Zhuravlev and Papoian [32], a consequence of the amplification of molecular noise of capping protein leading to macroscopic filopodial instability. The exact mechanisms remain to be elucidated.

The exact mechanisms of how filopodia promote cell-cell adhesion are controversial and unclear. Some groups suggest that cell-cell contact and signal-related events are initiated predominantly at the filopodia tips [33], [34]. A recent report contests the importance of filopodia for the formation of new cell-cell adhesions entirely [35].

Filopodia have been earlier described on neural progenitors [5], [36] and they have been ascribed to assist migration of NSCs/neurospheres in vitro, where it has been proposed that beating filopodia propel the neursopheres [2] by transiently adhering and then detaching from the underlying substrate [5]. Their role in cell-cell adhesion of NSCs has hitherto been unexplored. Filopodia on neural progenitors have various proteins and signalling molecules some of which appear to be involved in neurogenesis during development [37]. In mammalian cells each individual filopodium is made up of a cylindrical plasma membrane extension enclosing a tight bundle of 15–20 linear actin filaments all oriented in parallel, with their barbed ends distal from the cell body [38]. In addition to actin filaments a number of proteins and cell adhesion molecules (CAMs) are associated with filopodia, such as cadherins, integrins, NCAMs (neural cell adhesion molecules), WASP (Wiskott-Aldrich syndrome protein), formins, myosins, and facsins [39]. Rat neurosphere NSCs have been shown to express a variety of CAMs including E- and N-cadherin, α- and β-catenins [40]. Cadherins are the probable candidates for anchoring NSCs in stem cell niches [41]. They also appear to have other functions such as the regulation of NSC proliferation in rodents [40]. Cadherins are essential for initiating cell-cell contact, generally binding through homophilic interactions that are calcium-dependent; they drive cells to sort together to self-assemble into aggregates [42]. The adhesive patterns created by cadherin linkages, and their relative strengths, depend on factors such as the cell type, the 'age' of the contact, the type of cadherin molecules, the cytoplasmic proteins associated with the cadherins, and the architecture of the actin network [29]. Filopodial cell-cell adhesions that are activated by cadherin-cadherin binding, in general, converge via β-catenin on the Rho family of GTPases such as RhoA, Rac1, Cdc42 [43], [44] and eventually to the actin cytoskeleton. However, the specific mechanical and chemical cues that modulate the activity of the more prominent family members (RhoA, Rac1, and Cdc42) to each of the above mentioned processes is likely to vary between different cell types, receptors, or stimuli. Integrins are differently distributed in different compartments of the hippocampal regions depending on the species, including humans, mice and rats [45]–[48]. Human neurospheres have been shown to express high levels of β1 integrins [49]. Although integrins primarily mediate cell-extracellular matrix adhesion, some integrins mediate cell-cell interaction through cell adhesion molecules such as NCAM or cadherins [50] and in fact there is significant feedback and crosstalk between the integrins and cadherins as well as the various Rho family members that further complicates the process [44], [50]. Both cadherins and integrins (calcium-dependent CAMs) as well as calcium-independent CAMs, such as NCAMs, are located on cell surface membranes [50]. The abrogation of both membrane-mediated as well as filopodial-mediated adhesion by a calcium ion chelator in our study indicates that calcium-dependent cell adhesion molecules such as the cadherins and/or integrins are likely involved in both types of adhesion processes.

All the receptors involved in the adhesion process eventually interact with the actin cytoskeleton to bring about the morphological changes involved in cell-cell adhesion. Cyt-D has been used to perturb the actin cytoskeleton. It binds to barbed ends of actin filaments, inhibiting both the association and dissociation of subunits at these ends, thus shortening the filaments [51]. At micromolar and sub-micromolar concentrations Cyt-D inhibits elongation at both ends of the actin filaments, thus increasing the dissociation rate of F-actin further [51], [52]. Experiments with Cyt-D revealed some unexpected facets of adhesion dynamics. Cyt-D, used at sub-micromolar concentrations in our study, shows a disruption of both filopodial and cytoskeletal actin of the NSCs. However, somewhat unexpectedly, it appears to have a differential effect in that it seems to lead to inhibition of filopodial adhesion but not membrane-membrane adhesion of the cells, thus implying the lack of actin cytoskeletal involvement in the latter condition. A possible hypothesis for this observation could be the formation of non-junctional contacts between appositional cell membranes of NSCs that would allow transmembrane adhesion proteins to interact without formation of junctional complexes and activation of the actin cytoskeletal assembly. This has been demonstrated for some integrins and cadherins, especially for cell locomotion as seen in the migrating tip of the axon [53]. Alternatively, cadherins may not function as homogenous populations at cell-cell contacts [54]. Cavey *et al*. [54] demonstrated that cadherin-actin interactions may in reality be more complex with two distinct pools of actin-one that is sensitive to an inhibitor of actin polymerization and another resistant to it. Cell-cell adhesion through E-cadherin redistribution upon actin disruption has been observed in primary epidermal keratinocytes [29]. Thus it is feasible that the structural organization and role of actin in cell-cell adhesion may vary depending upon the cell type and CAMs involved. This observation of ours needs to be properly accounted for and further work to dissect and identify the adhesion molecules and pathways involved is necessary. A combination of optical trapping and Raman spectroscopy, of the type very recently applied to stem cells [55] might prove to be of utility in such studies.

In addition to optical trapping, alternative techniques that may find utility in future experiments might include diffraction phase microscopy [56], atomic force microscopy in the liquid phase [24], and micropipette-based measurements [57]. It remains to be demonstrated whether such techniques will afford at least the same temporal and spatial resolution employed in our optical trapping experiments. In the context of alternative experimental techniques, it is pertinent to note that NSCs readily adhere to uncoated glass surfaces as well as to surfaces coated with proteins like agarose. The optical trap technique, when combined with a flow cell, offers a real advantage as it enables single cells to be studied while they are kept away from surfaces by optical forces.

In summary, we have utilized an optical trapping method in conjunction with a fluid flow cell to quantitatively explore the spatio-temporal dynamics of cell-cell and cell-neurosphere adhesion. Hitherto unavailable information has been obtained on the most probable minimum time (∼5 s) and most probable minimum distance of approach (4–6 µm) required for irreversible adhesion of proximate cells to occur. Our experiments also allow us to study and quantify the spatial characteristics of filopodial- and membrane-mediated adhesion, and to probe the functional dynamics of NSCs to quantify a lower limit of the adhesive force by which NSCs aggregate (∼18 pN). Our findings, which we have also validated by computational modeling, have important implications for the neurosphere assay: once aggregated, neurospheres cannot be disassembled by vigorous shaking or by thermal effects. Our results provide quantitative affirmation to doubts expressed in the literature (see [1], [2] and references therein) about the neurosphere assay's validity as an accurate measure of clonality and “stemness”. Post-adhesion dynamics were also studied and oscillatory motion in filopodia-mediated adhesion was observed. Furthermore, we have also explored the effect of the removal of calcium ions: both filopodia-mediated as well as membrane-membrane adhesion were inhibited. On the other hand, F-actin disrupted the dynamics of such adhesion events such that filopodia-mediated adhesion was inhibited but not membrane-membrane adhesion. Our work also clearly brings out the utility of the optical trapping technique in helping develop new, quantitative insights into cell adhesion dynamics in such manner wherein results obtained in single-cell experiments begin to have relevance to adhesion phenomena in the macroscopic domain, such as in the neurosphere assay.

## Supporting Information

Figure S1 **Characterization of adult rat hippocampal neural progenitors in culture.** A) Immunofluorescence staining of undifferentiated neurospheres/neural progenitors for I) nestin and musashi, II) O4 and III) Sox-2. B) Proliferation of neural progenitors in culture demonstrated by BrDU incorporation and anti-BrDU antibody immunofluorescence staining. C) Demonstration by immunofluorescent staining of neural progenitor differentiation to I) mature neuronal cells (Neu-N), II) oligodendroglial cells (O1), III) astrocytic cells (GFAP).
(TIF)

Text S1 **Calculations of forces experienced by NSCs in vitro.**
(DOC)

Movie S1 **A real-time movie depicting the adhesion of two NSCs by a filopodial bridge when brought into contact for ∼5 secs.** Adhesion is confirmed when on moving the trapped cell (trap focus is marked by a white cross) the adhered cell moves along with it.
(AVI)

Movie S2 **A real-time movie depicting the adhesion of two NSCs through membrane-membrane interaction.** Adhesion is confirmed when the trapped cell does not move with trap focus (marked by X) as it is adhered to the other cell, while the latter is attached to the coverslip.
(AVI)

Movie S3 **A real-time movie depicting the adhesion of a NSC to a neurosphere through a filopodial interaction when brought into contact for more than 5 secs.** In this case again, adhesion is confirmed when the trapped cell does not move with the trap focus, as it attached to the neurosphere which cannot be manipulated by the optical trap.
(AVI)

Movie S4 **A real-time movie depicting the adhesion of a NSC with a neurosphere through membrane interactions when brought into contact for more than 5 secs.** Adhesion is observed in the same way.
(AVI)

Movie S5 **A real-time movie showing the initial adhesion of two neurospheres at contact points, at ∼4 sec followed finally by complete fusion to form a single neurosphere at ∼40 s.**
(AVI)

Movie S6 **A real-time movie showing the absence of adhesion between EGTA-treated NSCs brought into close contact by surface membranes at 6 s.**
(AVI)

Movie S7 **A real-time movie showing the interaction of a single NSC with a neurosphere after Cyt-D treatment.** Here again the cell is kept in contact with the neurosphere for a period of ∼5 sec. The NSC adheres to the neurosphere by membrane-membrane adhesion but not by the visible filopodium.
(AVI)

Movie S8 **A representative real-time movie showing the interaction of a single NSC with the same neurosphere as in Movie 6 after 0.1 µM Cyt-D treatment.** In spite of keeping the cell in contact with a filopodium on the neurosphere surface for a longer period of time, i.e. ∼8 s, no adhesion is seen to occur and the cell moves back along with the trap focus.
(AVI)

Movie S9 **A 3-D reconstruction of the F-actin filament-stained normal NSC.** Note the presence of rich F-actin staining in the filopodia and cytoskeleton.
(AVI)

Movie S10 **A 3-D reconstruction of an F-actin-stained NSC treated with 0.1 µM Cyt-D.** Note, the absence of any F-actin staining in the filopodia and a reduced, granular staining of cytoskeletal F-actin.
(AVI)
