# Supplementary figures and images for: Assembling Neurospheres: Dynamics of Neural Progenitor/Stem Cell Aggregation Probed Using an Optical Trap
Source: PLoS One. 2012 Jun 5;7(6):e38613. doi: 10.1371/journal.pone.0038613 (PMC3367915; doi:10.1371/journal.pone.0038613)

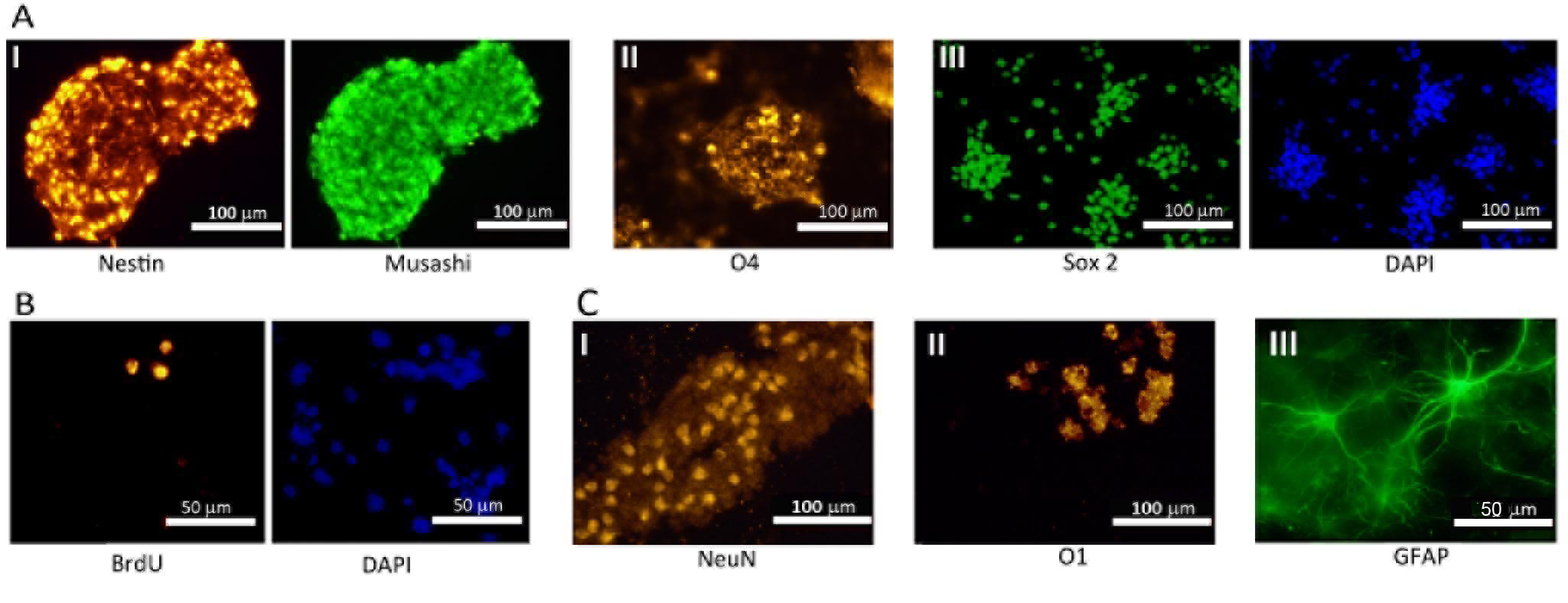

Supplement: Figure S1 — Characterization of adult rat hippocampal neural progenitors in culture. A) Immunofluorescence staining of undifferentiated neurospheres/neural progenitors for I) nestin and musashi, II) O4 and III) Sox-2. B) Proliferation of neural progenitors in culture demonstrated by BrDU incorporation and anti-BrDU antibody immunofluorescence staining. C) Demonstration by immunofluorescent staining of neural progenitor differentiation to I) mature neuronal cells (Neu-N), II) oligodendroglial cells (O1), III) astrocytic cells (GFAP). (TIF) [file pone.0038613.s001.tif]
