# Supplementary material for: Assembling Neurospheres: Dynamics of Neural Progenitor/Stem Cell Aggregation Probed Using an Optical Trap
Source: PLoS One. 2012 Jun 5;7(6):e38613. doi: 10.1371/journal.pone.0038613 (PMC3367915; doi:10.1371/journal.pone.0038613)
Supplement: Text S1 — Calculations of forces experienced by NSCs in vitro. (DOC) [file pone.0038613.s002.doc]

**Supplementary Material**

**Text T1: Calculation of forces experienced by NSCs *in vitro***

We have carried out computations to estimate the forces experienced by NSCs in vitro using two different models. In one model we take cognizance of fluid dynamics forces and in the second approach we adopt a purely kinetic approach in which temperature determines the Brownian motion of NSCs suspended in a fluid.

1. **Calculations using the fluid dynamics model**

**A**

**
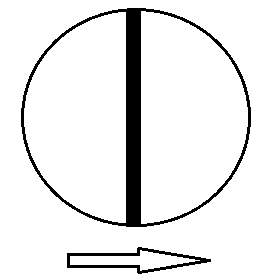
**

**B C**


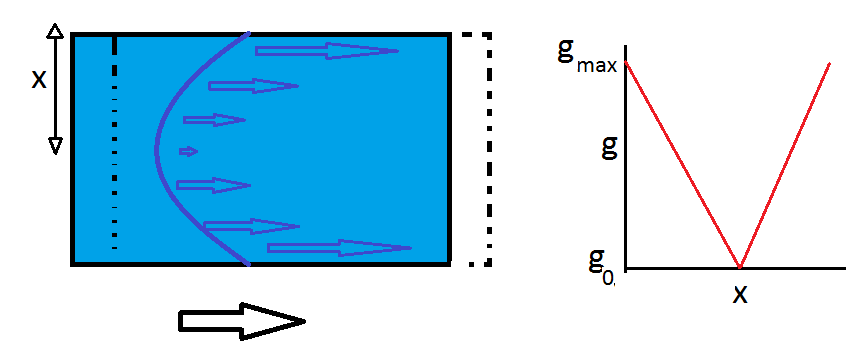


We assume that the culture dish containing the NSCs is rectangular and has infinite length. Such an assumption allows the calculation of shear forces in one dimension. Such an assumption will hold valid for the cells lying along the diameter perpendicular to the direction of the shaking of the plate as shown in panel A above. However, the results will be valid only for cells located within the central area of the rectangular plate where the one-dimensional approximation is most likely to be valid (see main body of text).

When the culture dish is shaken, a pair of adhered NSCs may separate because of the shear that is created. Panel B depicts the movement of dish towards the right of the screen; such movement would give rise to flow velocities whose magnitudes are depicted by the violet arrows in Panel B.

We denote X as the location of an NSC from the edges of the culture dish. The gradient (g) of the flow velocity pattern decreases linearly, and becomes zero at the midpoint of the dish (see Panel C). Hence, the shear force at the centre of the dish is always zero and it increases towards the edges of the plate. Thus, the adhered NSCs will not separate at the center, but their propensity to separate increases towards the edges. By estimating a value for g­_max_, it becomes possible to predict the distance from the centre of the dish that an adhered NSC has to be located at to experience shearing forces of more than 10 pN.

Determination of g_max_ is as follows:

Assume that the dish is shaken to and fro at a frequency h (Hz) with amplitude s/2 (m). Also, assume that the dish weighs w (kg).

To complete half an oscillation (that is, when the dish traverses from one extreme position to the other), the distance it travels is s, and the time taken is half a time period, that is, 1/(2h). Therefore, assuming that the dish accelerates in continuous fashion, with an acceleration a between these two points,

s = ½ * a *(1/(2h))^2^.

Thus, a = 8s*h^2^

Thus, the force (f) applied on the dish = w*a= **w*8s*h^2^.**

If this force is transmitted to the outermost layers of the fluid then,

F = ηAg_max_. where η is the dynamic viscosity, and A is the surface area.

Thus **g_max_= f/(**ηA)

g is zero at the center of the dish. If r denotes the width of the dish, then the value of g at a particular distance from the center will be

**g=(f/(ηA))*(R/r),** where R is the distance of a particular point from the centre of the plate.

When, R=r then g=g_max_

When R=0, g=0

The shear experienced by an adhered pair of NSCs will be due to the differences in the Stokes forces acting on the two cells. If we assume that the NSCs are, on average, D (m) in diameter, the velocity difference of the fluid impinging on the two NSCs would be Dg (m/s).

Then the difference in the Stokes force, and hence the shearing force, would be

6 *π* η (D/2)* Dg.

Therefore the shearing force acting on the two cells of the adhered NSC pair is given by:

**F= 6 *π***η***(D/2)*D*( w*8s*h^2^/(**η**A))*(R/r),**

where

η **= viscosity**

**D= diameter of NSC**

**h= shaking frequency**

**w= mass of dish**

**s= amplitude of shaking**

**A= cross sectional area of the culture medium**

**R= distance from the centre where the shear force is being measured**

**r = width of the dish.**

**Calculations for a 35 mm dish**

We use the following numbers:

η = 0.9 *10^-4^ Pa s

D= 10 * 10^-6^ m

h= 0.5 Hz

w= 0.007 kg

s= 0.005 m

A= 3.5 * 10^-4^ m^2^

R= distance from the centre where the shear force is being measured

r= 0.035 m

R at which the force is less than 10 pN = 1.9 mm

**Calculations for a 90 mm dish**

η = 0.9 *10^-4^ Pa s

D= 10 * 10^-6^ m

h= 0.5 Hz

w= 0.04 kg

s= 0.005 m

A= 9 * 10^-4^ m^2^

R= distance from the centre where the shear force is being measured

r= 0.09 m

R at which the force is less than 10 pN = 2 mm

**Calculations for a 6-well dish**

η = 0.9 *10^-4^ Pa s

D= 10 * 10^-6^ m

h= 0.5 Hz

w= 0.08 kg

s= 0.005 m

A= 3.5 * 10^-4^ m^2^

R= distance from the centre where the shear force is being measured

r= 0.035 m

R at which the shearing force is less than 10pN = 0.2 mm

2. **Calculations using a kinetic model**

In this model, the culture dish may assume any shape and the calculations are valid for cells lying in any part of the dish.

We assume that the temperature is T, yielding a Brownian energy, K_B_T.

We assume that the NSCs adhered as a pair use their entire Brownian energy to move in a straight line, with each of the two cells moving in mutually opposite directions. Such motion is generally referred to as ballistic motion. Such a motion is limited to very small distances (<1 nm).

The Stokes force during such motion = 6 *π* η *(D/2)*v

where η = viscosity, D = diameter of cell, v = velocity of ballistic motion.

To become separated, each cell in the pair would have to move a distance d.

Thus, the energy spent on movement though the fluid = 6 *π* η *(D/2)*v*d

Since Brownian energy = Energy spent on movement,

K_B_T = 6 *π* η *(D/2)*v*d

Thus, the maximum velocity that can be achieved is

v= K_B_T / (6 *π* η *(D/2)*d)

We consider two cells moving away from each other at a velocity v; the total relative velocity is 2v. This velocity is converted into a force on the bonds that contribute to cell adhesion when the bonds are fully stretched and each cell is brought to a rest (see the diagram below).


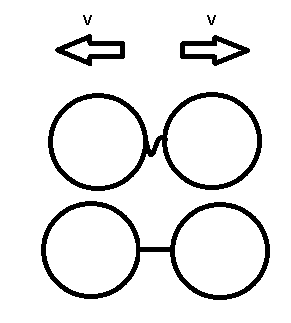


In case of the upper panel, no force is transmitted to the adhesion molecules at the junction, whereas, in the lower panel, as the adhesion molecules are stretched, a force is transmitted to them. For maximum force to be transmitted to the adhesion molecules, the cells must come to rest.

We consider that the cells take time t to come to rest and the bonds to simultaneously be completely stretched.

The accelaration of each of the cells is denoted by 2v/t.

Therefore, the force exerted on the adhesion molecules is w* 2v/t, where w= mass of each cell.

Thus the maximum force due to Brownian motion is

**f= (2w/t)*** **K_B_T / (6 *π*** η ***(D/2)*d),**

where

w= cellular mass= 10^-14^ kg

T = temperature in Kelvin= 310 K

K_B_ = 1.3806488 ×10^−23^

η = media viscosity = 0.9 *10^-4^ Pa s

D = cellular diameter = 10 ^-5^ m

d = length of intercellular bonds = 10 * 10^-9^ m (see text of the main paper.)

t= 1 s (see text of the main paper)

Thus, f= 10 *10^-19^ N = 10^-6^ pN

The maximum amount of force is generated when the bond is stretched to breaking point and the cells are brought to a halt. If we assume that the adhesion takes ~1 s to break, the maximum force that can be generated will be 8 orders of magnitude less than the required force for disassembly of the adhered NSCs.

Even if the temperature is increased by 50-60 C, no major change in the force regime can be perceived as temperature is linearly proportional to the force generated.
